# Supplementary material for: Assessing cardiovascular disease risk and social determinants of health: A comparative analysis of five risk estimation instruments using data from the Eastern Caribbean Health Outcomes Research Network
Source: PLoS One. 2025 Jan 24;20(1):e0316577. doi: 10.1371/journal.pone.0316577 (PMC11760610; doi:10.1371/journal.pone.0316577)
Supplement: S1 Table — Values highlighted in bold signify a statistically significant difference at the 5% level between the full sample and study sample. (DOCX) [file pone.0316577.s001.docx]

Supplementary Table 1. Sensitivity analysis showing demographic characteristics of full sample (n=2575) vs those with complete data (n=1777)

|  | **Full sample** | **Study sample** |
| --- | --- | --- |
| **Age in years (mean, 95% CI)** | **57.3 (56.9, 57.7)** | **55.4 (55.0, 55.8)** |
| **Women (proportion, 95% CI)** | 65.2 (63.5, 67.0) | 64.3 (62.0, 66.5) |
| **Education (proportion, 95% CI)** |  | |
| Less than high school | **36.1 (34.4, 37.9)** | **30.6 (28.4, 32.8)** |
| High school graduate | 23.8 (22.3, 25.4) | 23.1 (21.2, 25.2) |
| Associates degree/some college | 21.7 (20.2, 23.2) | 23.8 (21.8, 25.8) |
| College degree | **18.3 (16.9, 19.7)** | **22.5 (20.6, 24.5)** |
| **Occupation (proportion, 95% CI)** |  | |
| Professional | 30.3 (28.3, 32.3) | 33.8 (31.2, 36.4) |
| Semi-professional | 50.4 (48.2, 52.6) | 48.7 (46.0, 51.5) |
| Non-professional | 19.3 (17.7, 21.1) | 17.5 (15.5, 19.7) |

Values highlighted in **bold** signify a statistically significant difference at the 5% level between the full sample and study sample
