# Supplementary material for: Assessing cardiovascular disease risk and social determinants of health: A comparative analysis of five risk estimation instruments using data from the Eastern Caribbean Health Outcomes Research Network
Source: PLoS One. 2025 Jan 24;20(1):e0316577. doi: 10.1371/journal.pone.0316577 (PMC11760610; doi:10.1371/journal.pone.0316577)
Supplement: S2 Table — (DOCX) [file pone.0316577.s002.docx]

Supplementary table 2. Baseline 10-year CVD risk categorization in the ECHORN cohort according to five different risk estimator tools

|  | Framingham non-lab | | | Framingham lab | | | AHA/ASCVD | | | WHO lab | | | WHO non-lab | | |
| --- | --- | --- | --- | --- | --- | --- | --- | --- | --- | --- | --- | --- | --- | --- | --- |
|  | CVD Low Risk | CVD Int. Risk | CVD High Risk | CVD Low Risk | CVD Int. Risk | CVD High Risk | CVD Low Risk | CVD Int. Risk | CVD High Risk | CVD Low Risk | CVD Int. Risk | CVD High Risk | CVD Low Risk | CVD Int. Risk | CVD High Risk |
| Overall | 40% | 30% | 31% | 50% | 27% | 22% | 53% | 29% | 19% | 74% | 21% | 4% | 78% | 21% | 2% |
| Male | 19% | 34% | 47% | 32% | 31% | 37% | 39% | 36% | 25% | 69% | 25% | 7% | 71% | 25% | 4% |
| Female | 50% | 27% | 23% | 60% | 25% | 14% | 61% | 24% | 15% | 77% | 19% | 3% | 81% | 19% | 0% |
| Age (40-49) | 77% | 19% | 4% | 86% | 11% | 3% | 92% | 7% | 1% | 99% | 1% | 0% | 99% | 1% | 0% |
| Age (50-59) | 39% | 38% | 23% | 53% | 33% | 15% | 67% | 27% | 6% | 88% | 10% | 2% | 93% | 7% | 0% |
| Age (60-69) | 11% | 31% | 58% | 21% | 36% | 42% | 21% | 55% | 25% | 49% | 44% | 7% | 53% | 45% | 2% |
| Age (70+) | 2% | 20% | 77% | 9% | 30% | 60% | 0% | 25% | 75% | 2% | 70% | 28% | 1% | 85% | 14% |
