# Supplementary material for: Assessing cardiovascular disease risk and social determinants of health: A comparative analysis of five risk estimation instruments using data from the Eastern Caribbean Health Outcomes Research Network
Source: PLoS One. 2025 Jan 24;20(1):e0316577. doi: 10.1371/journal.pone.0316577 (PMC11760610; doi:10.1371/journal.pone.0316577)
Supplement: S3 Table — (DOCX) [file pone.0316577.s003.docx]

Supplementary table 3. Agreement between high-risk categories of five cardiovascular risk estimator tools in the ECHORN cohort

|  |  | **Framingham lab** | |
| --- | --- | --- | --- |
|  |  | Lower risk | High risk |
| **Framingham non-lab** | Lower risk | 1221 | 20 |
|  | High risk | 174 | 362 |
|  | *Kappa (95%CI)* | *0.72 (0.68, 0.75)* | |
|  |  | **AHA/ASCVD** | |
|  |  | Lower risk | High risk |
| **Framingham non-lab** | Lower risk | 1237 | 4 |
|  | High risk | 318 | 218 |
|  | *Kappa (95%CI)* | *0.48 (0.44, 0.53)* | |
|  |  | **WHO lab** | |
|  |  | Lower risk | High risk |
| **Framingham non-lab** | Lower risk | 1241 | 0 |
|  | High risk | 467 | 69 |
|  | *Kappa (95%CI)* | *0.18 (0.14, 0.21)* | |
|  |  | **WHO non-lab** | |
|  |  | Lower risk | High risk |
| **Framingham non-lab** | Lower risk | 1241 | 0 |
|  | High risk | 513 | 23 |
|  | *Kappa (95%CI)* | *0.06 (0.04, 0.08)* | |
|  |  | **AHA/ASCVD** | |
|  |  | Lower risk | High risk |
| **Framingham lab** | Lower risk | 1380 | 15 |
|  | High risk | 175 | 207 |
|  | *Kappa (95%CI)* | *0.63 (0.58, 0.67)* | |
|  |  | **WHO lab** | |
|  |  | Lower risk | High risk |
| **Framingham lab** | Lower risk | 1395 | 0 |
|  | High risk | 313 | 69 |
|  | *Kappa (95%CI)* | *0.27 (0.22, 0.31)* | |
|  |  | **WHO non-lab** | |
|  |  | Lower risk | High risk |
| **Framingham lab** | Lower risk | 1394 | 1 |
|  | High risk | 360 | 22 |
|  | *Kappa (95%CI)* | *0.09 (0.06, 0.12)* | |
|  |  | **WHO lab** | |
|  |  | Lower risk | High risk |
| **AHA/ASCVD** | Lower risk | 1552 | 3 |
|  | High risk | 156 | 66 |
|  | *Kappa (95%CI)* | *0.43 (0.36, 0.50)* | |
|  |  | **WHO non-lab** | |
|  |  | Lower risk | High risk |
| **AHA/ASCVD** | Lower risk | 1553 | 2 |
|  | High risk | 201 | 21 |
|  | *Kappa (95%CI)* | *0.16 (0.10, 0.22)* | |
|  |  | **WHO non-lab** | |
|  |  | Lower risk | High risk |
| **WHO lab** | Lower risk | 1703 | 5 |
|  | High risk | 51 | 18 |
|  | *Kappa (95%CI)* | *0.38 (0.25, 0.50)* | |
