# Supplementary material for: Assessing cardiovascular disease risk and social determinants of health: A comparative analysis of five risk estimation instruments using data from the Eastern Caribbean Health Outcomes Research Network
Source: PLoS One. 2025 Jan 24;20(1):e0316577. doi: 10.1371/journal.pone.0316577 (PMC11760610; doi:10.1371/journal.pone.0316577)
Supplement: Attachment — Submitted filename: Response to Reviewers.docx [file pone.0316577.s004.docx]

November 25, 2024

Dear *PLOS ONE* editorial staff,

We appreciate the thoughtful review of our manuscript. Herein, please find our rebuttals to each reviewer comment, in *italics*.

Sincerely,

Jeremy Schwartz

Reviewer #1

1. Are the "significant discrepancies in CVD risk assessment across tools" similarly discrepant in other cohorts or more/less discrepant in the ECHORN cohort?

*As we write in lines 105-107, these different risk assessment tools consistently demonstrate discrepancies. In this study, we did not seek to quantify the degree of discrepancy in ECHORN when compared to other cohorts.*

2. Table 4, Lines 401-402: I admittedly go back and forth with regards to wanting an analysis similar to Table 4 between participants who identified as Black and Other (including White in Other here given lower n numbers).. Or having a model that includes race/ethnicity with the age and sex adjustments if statistically appropriate. It may be somewhat unaligned with the described goal to develop "race- and ethnicity-free CVD risk estimates" but it also seems important to understand and interpret similarities or differences. I defer to the authors' expertise and preferences here.

*We appreciate the tension voiced by the reviewer, who is deferring to our expertise and preferences. First, it is difficult to define/assign race/ethnicity in multi-ethnic populations such as the ECHORN cohort, where mixed ancestry is common. Second, in this study, we chose to focus on SDoH rather than race as these directly influence lifestyle behaviors, stress levels, and access to preventative care, all of which contribute significantly to CVD risk. Race and ethnicity often act as proxies for these underlying inequities but do not capture the specific mechanisms driving risk.*

3. To note, BMI also has limitations in assessing cardiovascular risk in diverse populations ([https://pubmed.ncbi.nlm.nih.gov/26149446/](https://nam12.safelinks.protection.outlook.com/?url=https%3A%2F%2Fpubmed.ncbi.nlm.nih.gov%2F26149446%2F&data=05%7C02%7Cjeremy.schwartz%40yale.edu%7C559eece64f4444ed922008dd00130ba5%7Cdd8cbebb21394df8b4114e3e87abeb5c%7C0%7C0%7C638666805944178941%7CUnknown%7CTWFpbGZsb3d8eyJFbXB0eU1hcGkiOnRydWUsIlYiOiIwLjAuMDAwMCIsIlAiOiJXaW4zMiIsIkFOIjoiTWFpbCIsIldUIjoyfQ%3D%3D%7C0%7C%7C%7C&sdata=P7CQ1VPOjtkfNrLX7MweisKLRRMIQ%2FxXVOD8oSmL8Zc%3D&reserved=0)) and was curious how BMI categories here align with cardiovascular risk, understanding however that the focus here is on SDoH and that this can be a future study. Studies of cardiovascular health (rather than CVD risk) could also be a future study as it seems AHA Life's Simple 7 scores (but perhaps not Life's Essential 8) can be calculated from the ECHORN data.

*Our colleagues have previously published an analysis of the relationship between BMI and CVD risk in the ECHORN cohort: https://pubmed.ncbi.nlm.nih.gov/33632164/*.

4. Line 77, Line 331, Line 406: what determines "strong association" since food insecurity was associated with higher CVD risk in two out of the 5 risk tools (and high school graduate alone was associated in lower CVD risk for one out of the five tools)?

*We agree that this is better described as an association, rather than “strong”. We have removed the word “strong” from the text*

5. Line 78, Lines 405-406: did analyses really show association between lower educational attainment and increased CVD risk? or rather, i believe from Table 4, lower odds of high CVD risk for people with a college degree (compared to people with less than high school education)? i understand that these are similar, but they do not seem quite the same (especially in the relative absence of differences compared to reference group for the high school graduate and associate degree/some college groups).

*We agree and have amended the text so that it is clear that we are referring to the highest compared to the lowest level of education, rather than a gradient across all levels.*

6. Line 123: is this e.g. necessary or appropriately placed?

*Removed*

7. Lines 134-135: assuming these are both supposed to be em dashes, they appear to be different.

*Changed*

8. Line 147: semi-permanent (no space needed)

*There is not a space- unclear what the reviewer is referring to*

9. Line 154: audio-assisted (no space needed)

*There is not a space- unclear what the reviewer is referring to*

10. Line 157: A1c

*Removed altogether, as hemoglobin A1c was not part of the current analysis.*

11. Line 159: capitalize to align with other section headings

*Already is capitalized.*

12. Line 161: no need for comma

*Removed comma*

13. Line 174: is there a reason why A1c >=6.5% was not included here (since included in measurements per line 157)?

*We excluded A1c to bring our definition of diabetes in line with those used in population-based surveys in the US (NHANES) and globally (WHO STEPS). Agreed that it is confusing to mention A2c when we did not use it, so we have removed reference to it as per above.*

14. Line 195: Is there a reason why Globorisk (or Globorisk-LAC if available, [https://pubmed.ncbi.nlm.nih.gov/35711683/](https://nam12.safelinks.protection.outlook.com/?url=https%3A%2F%2Fpubmed.ncbi.nlm.nih.gov%2F35711683%2F&data=05%7C02%7Cjeremy.schwartz%40yale.edu%7C559eece64f4444ed922008dd00130ba5%7Cdd8cbebb21394df8b4114e3e87abeb5c%7C0%7C0%7C638666805944196804%7CUnknown%7CTWFpbGZsb3d8eyJFbXB0eU1hcGkiOnRydWUsIlYiOiIwLjAuMDAwMCIsIlAiOiJXaW4zMiIsIkFOIjoiTWFpbCIsIldUIjoyfQ%3D%3D%7C0%7C%7C%7C&sdata=RzLsjptSTjUMDhuwzZQLAYgEEpdXxD63gfTKZVUjcN0%3D&reserved=0)) was not included? somewhat answered in Lines 428-430..

*As noted by the reviewer, we do refer to Globorisk. However, we could not be exhaustive in our use of CVD risk calculators for practical reasons. We selected the included calculators based on informal conversations with local clinicians, who identified Framingham and ASCVD as the most commonly used calculators in clinical practice. We included the WHO tool due to its calibration using risk factor data from Caribbean populations, and the interest generated in it by local clinicians due to promotion of the tool in the region by WHO. The Globorisk-LAC is less well-known, and although it was derived using one Caribbean cohort, the overwhelming majority of the sample was from Latin America (only 3% of the sample is Caribbean).*

15. Lines 199, Line 202, Lines 376-378: It includes White, African American, and Other, correct?

*Please see response to next comment.*

16. Lines 207-208: why were participants who selected white classified as other and not white?

*The ASCVD tools is the only one from those selected for this study that requires race to be entered. It either designates participants as “African American” or “Other”. Note that the online tool allows one to select “White” also, but the underlying algorithm groups “White” and “Other” together. We have explained this in lines 205-206 (new line numbering after editing).*

17. Line 212: tools?

*Edited accordingly.*

18. Line 234: de-identified (no space needed)

*Edited accordingly.*

19. Line 261: suggest adding "in the full sample" to be clear

*Edited accordingly.*

20. Table 2: I'm assuming the total cholesterol and HDL were appropriately converted between mg/dL and mmol/L for the respective CVD risk tools?

*We confirm that we entered the measurements into the tools in the appropriate units.*

21. Table 3, Table 4, Supplementary Table 3: suggest ASCVD rather than AHA/ASCVD

*We have changed the text to ASCVD.*

21. Table 4: To my understanding, income relative to population was not measured rather perceived social status or subjective socioeconomic status. Advised changing the references to income here.

*This is our error and we have edited Table 4 accordingly.*

22. Table 4: suggest associate or associate's

*Edited accordingly.*

23. Line 333: [https://pubmed.ncbi.nlm.nih.gov/37648706/](https://nam12.safelinks.protection.outlook.com/?url=https%3A%2F%2Fpubmed.ncbi.nlm.nih.gov%2F37648706%2F&data=05%7C02%7Cjeremy.schwartz%40yale.edu%7C559eece64f4444ed922008dd00130ba5%7Cdd8cbebb21394df8b4114e3e87abeb5c%7C0%7C0%7C638666805944214745%7CUnknown%7CTWFpbGZsb3d8eyJFbXB0eU1hcGkiOnRydWUsIlYiOiIwLjAuMDAwMCIsIlAiOiJXaW4zMiIsIkFOIjoiTWFpbCIsIldUIjoyfQ%3D%3D%7C0%7C%7C%7C&sdata=EblKsEi8JViZcJIE2pWU0mTfXpLC5TaqdZqEn20hlzM%3D&reserved=0) compared the WHO instrument performance against another instrument, correct?

*Correct. This is one reference we have not included. We are not claiming that our paper is the first to use the new WHO instrument- but among the first.*

24. Lines 345-346: suggest a clearer and simpler word that disclaim and adding a reference

*On reconsideration, given conflicting data literature, we decided to remove this sentence altogether.*

25. Line 364: suggest COVID-19

*Edited accordingly.*

26. Line 399: ZIP

*Edited accordingly.*

27. Lines 399-400: including PREVENT (as it currently is) for analyses in non-US settings will not be possible since based on U.S. Postal Service ZIP code (and associated deprivation indices) though, correct? This sentence could be removed if desired or adapted.

*We edited to acknowledge this.*

28. Line 447: I don't see a "Research in Context" section?

*To our understanding, PLOS ONE does not require a Research in Context section. Editors can advise if otherwise.*

Reviewer #2

1. Line 227: The authors state that in their sensitivity analysis they compare demographics and outcomes among included and excluded participants. But the Supplementary Table 1 only includes data on some demographics and they do not include any data about outcomes of the study. And in line 432 they state that their sensitivity analysis demonstrate that missing data did not affect the risk score. As this is hard to believe (if they do not have data about these participants, they cannot calculate the risk scores, and unless they provide data on the incidence of Cardiovascular events or on disease prevalence among excluded individuals) they cannot assume that the sensitivity analysis discards bias in this study.

*We agree. The text has been changed to state that the sensitivity analyses showed that the missing data did not change the demographic characteristics pf the sample relative to the full cohort. We have removed the reference to comparison of outcomes. We acknowledge in the limitations that missing data may limit the strength of our findings.*

2. Methodology: It would be better if the authors describe more thoroughly the multivariable regression models they use (at least in the supplementary material) and the results of these models.

*We have added more information on the modeling strategy to the “Statistical Analyses” section.*

3. In the results displayed on table 4, there is a remarkable great range on the CI in the WHO non-lab model. This aspect deserves some explanation in the Discussion, as there is no such result in the Framingham non-lab model, despite they include roughly the same variables (except Blood-pressure treatment and diabetes).

*When the relationship between the independent and dependent variables is weak, the estimate's precision is lower, which can result in wide CIs. Unlike the Framingham non-lab, the WHO non-lab was not associated with any SDoH variable. This likely caused the wide CIs in table 4.*
4. In line 355 the authors state that reference 10 suggest that Framingham may underestimate CVD in Caribbean population, when they should speak about CVD risk.

*Noted and changed.*
